# Risk of Hyponatraemia in Cancer Patients Treated with Targeted Therapies: A Systematic Review and Meta-Analysis of Clinical Trials

**DOI:** 10.1371/journal.pone.0152079

**Published:** 2016-05-11

**Authors:** Rossana Berardi, Matteo Santoni, Silvia Rinaldi, Emilia Nunzi, Alessia Smerilli, Miriam Caramanti, Francesca Morgese, Mariangela Torniai, Agnese Savini, Ilaria Fiordoliva, Azzurra Onofri, Mirco Pistelli, Augusto Taccaliti, Stefano Cascinu

**Affiliations:** 1 Clinica di Oncologia Medica, Università Politecnica delle Marche, Azienda Ospedaliero-Universitaria Ospedali Riuniti Umberto I–GM Lancisi–G Salesi, Ancona, Italy; 2 Dipartimento Medicina Sperimentale, Università degli Studi di Perugia, Perugia, Italy; 3 Division of Endocrinology, A—Azienda Ospedaliero-Universitaria Ospedali Riuniti Umberto I–GM Lancisi–G Salesi, Ancona, Italy; University Campus Bio-Medico, ITALY

## Abstract

**Background:**

Hyponatraemia has been reported with targeted therapies in cancer patients. Aim of the study was to perform an up-to-date meta-analysis in order to determine the incidence and relative risk (RR) in cancer patients treated with these agents.

**Materials and Methods:**

The scientific literature regarding hyponatraemia was extensively reviewed using MEDLINE, PubMed, Embase and Cochrane databases. Eligible studies were selected according to PRISMA statement. Summary incidence, RR, and 95% Confidence Intervals were calculated using random-effects or fixed-effects models based on the heterogeneity of selected studies.

**Results:**

4803 potentially relevant trials were identified: of them, 13 randomized phase III studies were included in this meta-analysis. 6670 patients treated with 8 targeted agents were included: 2574 patients had hepatocellular carcinoma, whilst 4096 had other malignancies. The highest incidences of all-grade hyponatraemia were observed with the combination of brivanib and cetuximab (63.4) and pazopanib (31.7), while the lowest incidence was reported by afatinib (1.7). The highest incidence of high-grade hyponatraemia was reported by cetuximab (34.8), while the lowest incidences were reported by gefitinib (1.0). Summary RR of developing all-grade and high-grade hyponatraemia with targeted agents was 1.36 and 1.52, respectively. The highest RRs of all-grade and high-grade hyponatraemia were associated with brivanib (6.5 and 5.2, respectively). Grouping by drug category, the RR of high-grade hyponatraemia with angiogenesis inhibitors was 2.69 compared to anti-Epidermal Growth Factor Receptors agents (1.12).

**Conclusion:**

Treatment with biological therapy in cancer patients is associated with a significant increased risk of hyponatraemia, therefore frequent clinical monitoring should be emphasized when managing targeted agents.

## Introduction

Targeted therapies interfere with specific molecules involved in cancer cell growth, angiogenesis and survival, in contrast with traditional chemotherapy, drugs that mainly act against all actively dividing cells.

Such a different mechanism of action explains the absence of adverse events traditionally observed with cytotoxic chemotherapy and the occurrence of new drug-related toxicity profiles.

Among serum electrolytes disorders, hyponatraemia is probably the most frequent biochemical alterations potentially related to the use of these new agents.

Although many cases are asymptomatic, hyponatraemia may cause neurological symptoms, particularly when serum sodium declines rapidly or by a substantial extent [1].

Furthermore literature data suggest that hyponatraemia can be considered an unfavourable prognostic factor in this setting and it has been also hypothesized to adversely affect the response to anticancer treatment [2,3].

Moreover an effective and timely normalization of sodium levels could lead to a positive effect on prognosis of cancer patients.

The objective of the present study was to thoroughly assess incidence and relative risk of hyponatraemia in patients with solid tumors receiving targeted therapies through a revised meta-analysis of clinical trial in literature.

## Materials and Methods

### Selection of Studies

This systematic review and meta-analysis was achieved adhering to PRISMA guidelines for clinical trial selection [4]. PubMed and MEDLINE (since January 1966), Embase (since 1974) and the Cochrane Central Register of Controlled Trials (since 1967) quotations were revised in order to individuate studies of interest. In particular we selected more interesting trials generated from the research finding in Pubmed.

Searches were conducted entering combination of the keywords “cancer” or “solid tumor” associated to any of the following words: “abiraterone”, “afatinib”, “aflibercept”, “axitinib”, “bevacizumab”, “brivanib”, “cabozantinib”, “cediranib”, “cetuximab”, “crizotinib”, “dabrafenib”, “dovitinib”, “enzalutamide”, “erlotinib”, “everolimus”, “figitumumab”, “gefitinib”, “icotinib”, “imatinib”, “ipilimumab”, “lapatinib”, “linifanib”, “neratinib”, “nilotinib”, “nivolumab”, “orteronel”, “panitumumab”, “panobinostat”, “pazopanib”, “pembrolizumab”, “pertuzumab”, “ramucirumab”, “regorafenib”, “sorafenib”, “sunitinib”, “T-DM1”, “temsirolimus”, “tivozanib”, “trastuzumab”, “tremelimumab”, “vandetanib”, “vemurafenib”. We evaluated exclusively human studies in English literature that met the requirements listed below: (1) prospective randomized phase III trials enrolling patients affected by solid tumors; (2) patients randomly assigned to treatment arm (targeted agents) or control arm (standard of care, best supportive care or placebo) and (3) provided records regarding treatment-related and non-tumor associated hyponatraemia.

Full articles were obtained, and we checked for additional appropriate references. Where results were reported or updated in two or more publication, we selected the most recent or most thorough.

The primary objective of our study was to assess whether there is a correlation between hyponatraemia and treatment with targeted therapy.

Comparative trials presenting targeted agents in both study arms were not considered, as well as numerous meta-analyses conducted in similar settings [5–16].

### Data Extraction and Quality Assessment

Data extraction was performed from full texts of eligible articles, by two independent evaluators (MS and EN). Data collected included author name list, year of publication, number of participants, treatment arms characteristic and targeted agent employed, number and grade (all-grade and high grade) of hyponatraemia cases reported in every arm.

National Cancer Institute’s Common Terminology Criteria for Adverse Events (CTCAE) version 2 or 3 were applied to define adverse events (AEs). Study quality and appropriateness of randomization, double-blinding, and withdrawals was determined basing on Jadad scoring system [17].

### Statistical Analysis

We considered the following summary measurements: incidence, relative risk (RR), and their corresponding 95% confidence intervals (CIs). Incidence evaluation was performed extracting from the safety section of eligible studies the following data: (1) number of patients receiving targeted therapy and (2) number of hyponatraemia cases. RRs of hyponatraemia was analyzed basing on data extracted from comparative trials in which patients were randomly assigned to receive targeted therapy or controls.

Cochran’s Q test was applied to all variables to detect statistical heterogeneity among study outcomes; inconsistency of effects was measured using the I2 index as a parameter of inconsistency across studies attributable to heterogeneity and chance. Homogeneity of variance was violated for p values <0.1. Basing on Cochran’s Q statistic significance it was decided whether to use random effects model (in case of significant Q test) or fixed effects model (in case of not significant Q test).

For each variable, model estimate and null hypothesis of overall non-significant difference between study- and control-group were tested. Microsoft Excel 2010 was employed to collect data; data analysis has been carried out with the “MATLAB and Statistics Toolbox Release 2012b”.

## Results

### Search Results

Four thousand, eight hundreds and three clinical trials studying target treatments employ in neoplastic patients resulted hypothetically significant to our research; of those, 2914 studies did not meet inclusion criteria due to any of the following causes: duplicate, phase I trials, not focused on targeted agents, reviews, observational studies, meta-analyses, case reports, letters or commentaries.

**Fig 1:**
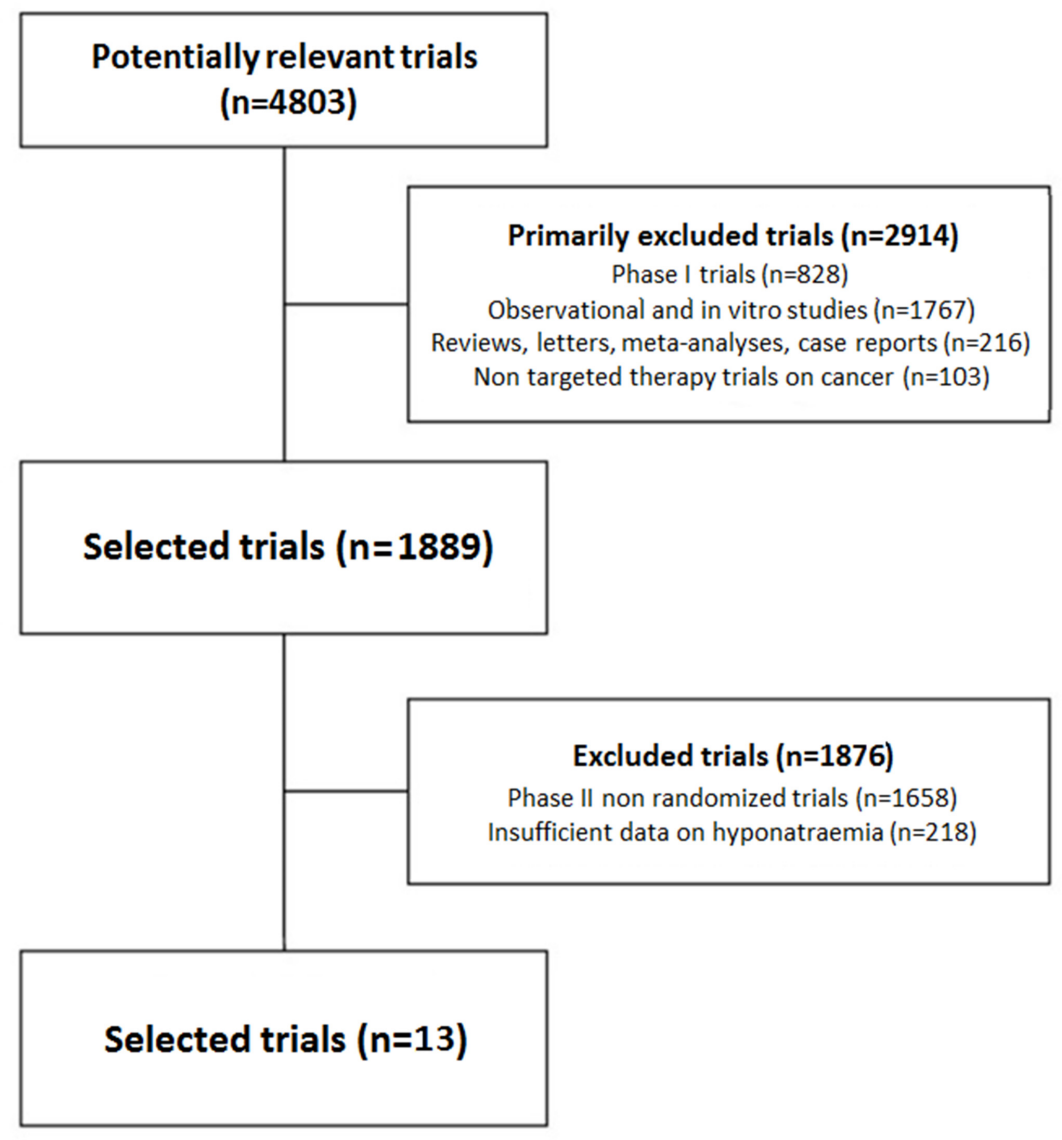
Selection of randomized controlled trials included in the meta-analysis according to PRISMA statement.

Of the remaining studies, 1658 were non-randomized phase II trials while 218 lacked Drug-related hyponatraemia records in the safety profile. Eventually 13 trials [18–30] were judged suitable and relevant for our study. The selection process of studies is represented in Fig 1. Baseline features of included trials are listed in Table 1.

**Table 1:** Baseline characteristics of randomized trials included in the meta-analysis. In grey the studies excluded from the relative risk (RR) analysis due to the presence of an active control arm.

Author and Year	Ref.	Phase	Malignancy	Treatment		N. subjects		Jadad scale
				Targeted therapy	Control Arm	Targeted therapy	Control Arm	
Wu *et al*. 2014	18	3	NSCLC	Afatinib	CDDP + GEM	239	113	3
Llovet *et al*. 2013	19	3	HCC	Brivanib	Placebo	263	132	4
Johnson *et al*. 2013	20	3	HCC	Brivanib	Sorafenib	575	575	4
Siu *et al*. 2013	21	3	Colorectal cancer	Brivanib + Cetuximab	Cetuximab	372	373	3
Burtness *et al*. 2005	22	3	HN tumors	CDDP + Cetuximab	CDDP	58	58	5
Lordick *et al*. 2013	23	3	Gastric cancer	CDDP + Capecitabine + Cetuximab	CDDP + Capecitabine	446	436	3
Crosby *et al*. 2013	24	2/3	Oesophageal cancer	Chemoradiotherapy +Cetuximab	Chemoradiotherapy	129	129	3
Gaafar *et al*. 2011	25	3	NSCLC	Gefitinib	Placebo	85	86	4
Argiris *et al*. 2013	26	3	HN tumors	DTX + Gefitinib	DTX	124	129	4
Cainap *et al*. 2015	27	3	HCC	Linifanib	Sorafenib	510	519	3
Sternberg *et al*. 2010	28	3	RCC	Pazopanib	Placebo	290	145	4
Flaherty *et al*. 2013	29	3	Melanoma	CBP + PTX + Sorafenib	CBP + PTX	393	397	4
Ramalingam *et al*. 2010	30	2	NSCLC	CBP + PTX + Vorinostat	CBP + PTX	62	32	3

Legend. CBP: Carboplatin, CDDP: Cisplatin, DTX: Docetaxel, GEM: Gemcitabine, HCC: hepatocellular carcinoma, HN: head and neck, NSCLC: non-small cells lung cancer, PTX: Paclitaxel, RCC: renal cell carcinoma.

### Quality of studies

The quality of trials selected for the meta-analysis was determined according to the Jadad assessment scale [31]. Follow-up time was appropriate in each trial. All the studies used either Common Terminology Criteria Adverse Events (CTCAE) version 2.0 or 3.0.

Jadad scores of the 13 studies included are reported in Table 1. The average of Jadad scores was 3.7 (range 3 to 5) standing for an adequate quality of the meta-analysis.

### Study Population

Study population accounted for 6670 participants of whom 2574 (39% of the total) were affected by hepatocellular carcinoma (HCC) [19,20,27], and 4096 affected by other malignancies (882 patients had gastric cancer [23], 790 patients had melanoma [29], 745 had colorectal cancer [21], 617 had non-small cell lung cancer (NSCLC) [18,25,30], 435 had renal cell carcinoma (RCC) [28], 369 had head and neck tumors (HN) [22,26] and 258 had oesophageal cancer [24]).

An Eastern Cooperative Oncology Group performance status (ECOG-PS) not higher than 2 was required for enrolment in all 13 studies along with fair renal and hepatic functions, coagulation and haematological parameters. Baseline features of included trials are listed in Table 1. Selected target agents and their mechanism of action are listed in Table 2.

**Table 2:** Evaluated target agent for hyponatremia and their mechanism of action.

DRUG	MECHANISM OF ACTION
**AFATINIB**	Irreversible covalent inhibitor of the receptor tyrosine kinases EGFR and erbB-2 (HER2)
**BRIVANIB**	VEGFR2 inhibitor
**CETUXIMAB**	Chimeric (mouse/human) monoclonal antibody inhibiting EGFR
**GEFITINIB**	Elective inhibitor of EGFR tyrosine kinase domain
**LINIFANIB**	Multi-targeted receptor inhibitor of VEGFR, PDGFR and CSF-1R
**PAZOPANIB**	Selective multi-targeted receptor tyrosine kinase inhibitor (c-KIT, FGFR, PDGFR and VEGFR)
**SORAFENIB**	Small multikinase inhibitor (VEGFR, PDGFR and Raf family kinases)
**VORINOSTAT**	HD inhibitor

Legend. EGFR: Epidermal Growth Factor Receptor, VEGFR: Vascular Endothelial Growth Factor Receptor, PDGFR: platelet-derived growth factor receptor, CSF-1R: colony stimulating factor 1 receptor, FGFR: fibroblastic growth factor receptor, HDI: Histone Deacetylase inhibitor.

### Incidence of all-grade and high-grade hyponatraemia

Occurrences of hyponatraemia (all-grade and high-grade) accounted for a total of 1402 cases; of those 575 all-grade events occurred among the 3036 patients belonging to treatment groups versus 284 among controls. Considering only patients assigned to receive targeted therapy, the incidences of all-grade and high-grade hyponatraemia were 25.6% (95% CI 23.8 to 27.4) and 10.0% (95% CI 9.1 to 11.0), respectively.

All-grade hyponatraemia reached its maximum incidence with the combination of brivanib and cetuximab [21] (63.4%, 95% CI 58.5 to 68.3) and with pazopanib [28] (31.7%, 95% CI 26.3 to 37.1), whereas afatinib [18] (1.7%, 95% CI 0.0 to 3.0) showed the lowest incidence of hyponatraemia. However, several studies did report only high-grade events [22,24–26,29].

Three hundred and fifty six high-grade hyponatraemia occurrences were reported in patients assigned to treatment arms and 187 in the controls. The highest and lowest incidences of high-grade hyponatraemia were observed with cetuximab [22] (44.8%, 95% CI 32.0 to 57.6), and gefitinib [26] (1.0%, 95% CI 0 to 2.3), respectively. The incidences of all-grade and high-grade hyponatraemia are reported in Table 3.

**Table 3:** Incidence of all-grade and high-grade hyponatraemia by individual study. In grey the studies excluded from the relative risk (RR) analysis due to the presence of an active control arm.

Author and Year	Ref.	Treatment		N. all grade events/ subjects		N. high-grade events/subject		Incidences of all-grade hyponatraemia with targeted therapy (95% CI)	Incidences of high-grade hyponatraemia in the control arm (95% CI)
		Targeted therapy	Control Arm	Targeted therapy	Control Arm	Targeted therapy	Control Arm		
Argiris *et al*. 2013	26	DTX + Gefitinib	DTX	Not reported	Not reported	1/124	4/129	Not reported	1.0 (1.0−2.4)
Burtness *et al*. 2005	22	CDDP + Cetuximab	CDDP	Not reported	Not reported	26/58	28/58	Not reported	44.8 (32.0−57.6)
Cainap *et al*. 2015	27	Linifanib	Sorafenib	Not reported	Not reported	19/510	17/519	Not reported	3.7 (2.1–5.4)
Crosby *et al*. 2013	24	Chemoradiotherapy +Cetuximab	Chemoradiotherapy	Not reported	Not reported	2/129	1/129	Not reported	15.5 (1.0−36.8)
Flaherty *et al*. 2013	29	CBP + PTX + Sorafenib	CBP + PTX	Not reported	Not reported	21/393	9/397	Not reported	5.3 (3.1−7.6)
Gaafar *et al*. 2011	25	Gefitinib	Placebo	Not reported	Not reported	14/85	7/86	Not reported	16.5 (8.6−24.4)
Johnson *et al*. 2013	20	Brivanib	Sorafenib	150/575	63/575	132/575	53/575	26.1 (22.5−29.7)	23.0 (19.5−26.4)
Llovet *et al*. 2013	19	Brivanib	Placebo	39/263	3/132	31/263	3/132	14.8 (10.5−19.1)	11.8 (7.9−15.7)
Lordick *et al*. 2013	23	CDDP + Capecitabine + Cetuximab	CDDP + Capecitabine	42/446	37/436	31/446	26/436	9.4 (6.7−12.1)	7.0 (4.6−9.3)
Ramalingam *et al*. 2010	30	CBP + PTX + Vorinostat	CBP + PTX	12/62	3/32	12/62	3/32	19.4 (9.5−29.2)	19.4 (9.5−29.2)
Siu *et al*. 2013	21	Brivanib + Cetuximab	Cetuximab	236/372	133/373	48/372	26/373	63.4 (58.5−68.3)	12.9 (9.5−16.3)
Sternberg *et al*. 2010	28	Pazopanib	Placebo	92/290	35/145	16/290	6/145	3170 (26.4−37.1)	5.5 (2.9−8.1)
Wu *et al*. 2014	18	Afatinib	CDDP + GEM	4/239	10/113	3/239	4/113	1.7 (0.0−3.3)	1.3 (0.0−2.7)

Legend. CBP: Carboplatin, CDDP: Cisplatin, DTX: Docetaxel, GEM: Gemcitabine, HCC: hepatocellular carcinoma, HN: head and neck, NSCLC: non-small cells lung cancer, PTX: Paclitaxel, RCC: renal cell carcinoma.

### RR of all-grade and high-grade hyponatraemia in the overall population and by single study

RR analysis was conducted considering 4 studies for the analyses of all-grade events [19,23,27,29] and 9 for high-grade events [19, 22–29]. In three studies placebo was administered in the control arm [19, 25,28], while patients in the other studies were assigned to active control arms [20,22–24,26,29,30].

**Fig 2:**
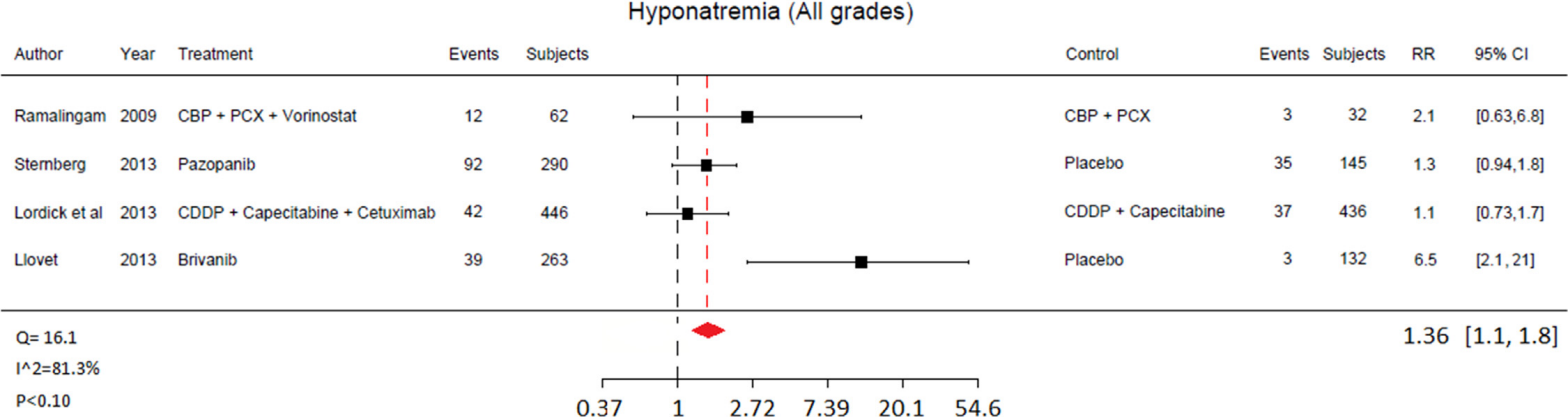
Relative Risk (RR) of all-grade hyponatraemia associated with targeted therapy by individual study.

**Fig 3:**
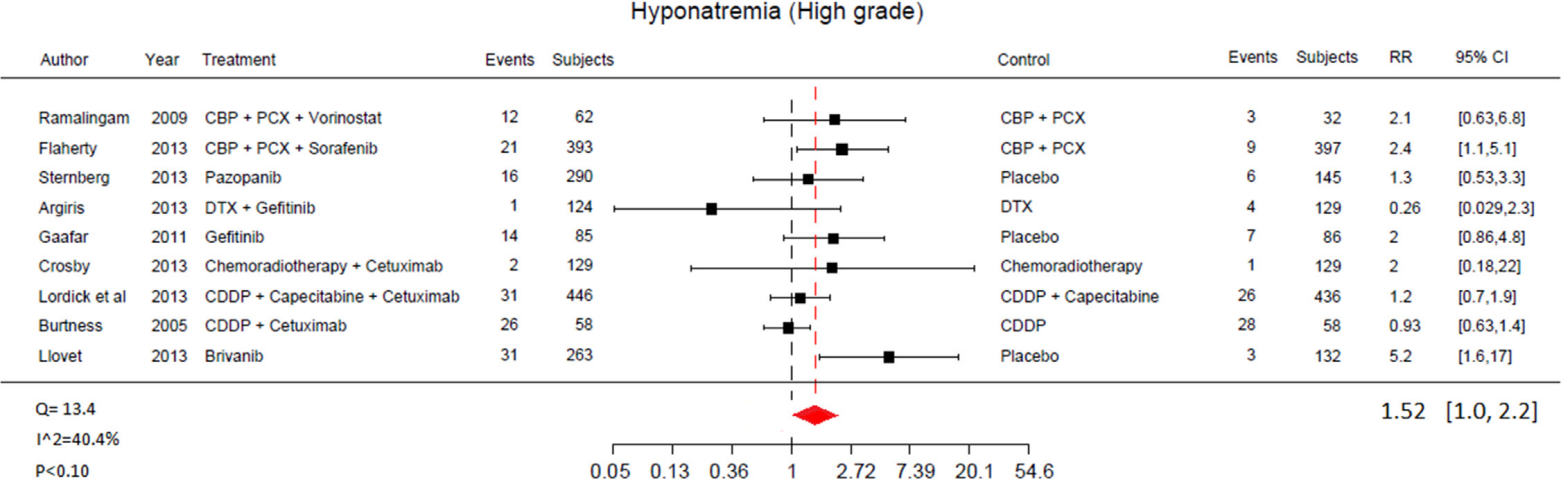
Relative Risk of high-grade hyponatraemia associated with targeted therapy by individual study.

In the overall study population, RR of all-grade and high-grade hyponatraemia was 1.36 (95% CI 1.06 to 1.75) for patients receiving targeted treatments compared to 1.52 (95% CI 1.06 to 2.20) in control arms. The RRs of all-grade hyponatraemia across selected trials are reported in Figs 2 and 3.

Eight different agents were available for this analysis. In patients stratified by single studies, the highest RR of all-grade hyponatraemia was associated with brivanib [19] (6.5, 95% CI 2.1 to 21.0). On the other hand, the lowest RR was associated with cetuximab [23] (1.1, 95% CI 0.73 to 1.70).

High-grade hyponatraemia highest RRs occurred with brivanib [19] (5.2; 95% CI 1.6 to 17.0), sorafenib [28] (2.4; 95% CI 1.1 to 5.1) and vorinostat [29] (2.1; 95% CI 0.63 to 0.81), while the lowest RR of high-grade hyponatraemia was observed with cetuximab [22] (0.93; 95% CI 0.63 to 1.42) and gefinitib [26] (0.26; 95% CI 0.03 to 2.33]. The RRs of all-grade hyponatraemia across selected trials are reported in Figs 2 and 3.

### RR of high-grade hyponatraemia by drug category

For an experimental examination, 5 out of the 6 targeted therapies studied in the RR analysis of high-grade hyponatraemia [19,22–28] were grouped into 2 categories: (1) inhibitors of angiogenesis (brivanib, pazopanib, sorafenib); (2) anti-Epithelial growth factor receptor tyrosine kinase inhibitors (EGFR-TKIs) or monoclonal antibodies (mAbs) (gefitinib, cetuximab). The same analysis was not performed for all-grade events due to the smaller number of studies available. Afatinib was omitted in reason of an active control arm [18]. Vorinostat was excluded due to the number of patients in this study [29], which was too small to constitute a single group.

**Fig 4:**
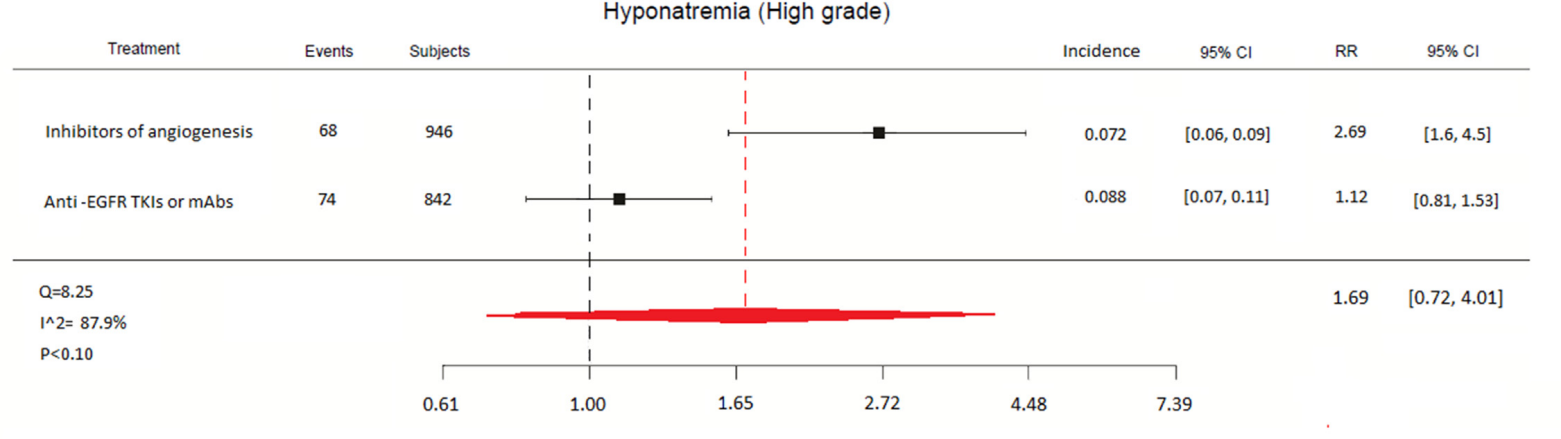
Relative Risk of high-grade hyponatraemia by drug category.

A total of 946 patients received inhibitors of angiogenesis [19,28,29], whereas 442 received anti-EGFR TKIs or mAbs [22–26]. The incidence of high-grade hyponatraemia was 7.2 [95% CI 5.5 to 8.8] with the inhibitors of angiogenesis and 8.8 [95% CI 6.9 11.0] with anti-EGFR TKIs or mAbs. Moreover, the RR of high-grade hyponatraemia with inhibitors of angiogenesis was 2.69 (95% CI 1.62 to 4.48) compared to anti-EGFR TKIs or mAbs (1.12 95% CI 0.81 to 1.53) (Fig 4).

### Publication Bias

Publication biases were quantified by Egger and Begg tests for both the incidence and RR. Egger test showed z = 1·62 *p* = 0·11, while Begg test showed Kendall's tau = 0.1111 *p* = 0.7614. Funnel plots are showed in S1 Fig.

## Discussion

Hyponatraemia represents an increasingly important issue in oncology clinical practice since it negatively correlates with performance status and with prognosis of cancer patients [32]. Patients with hyponatraemia have a higher risk of mortality and present a longer time of hospitalization with consequent cost increases [33]. An early detection and a prompt treatment of this disorder could prevent serious neurologic complication and improve overall survival (OS) [34]. For this reasons, it is pivotal for both physicians and patients to be aware about the risk of drug-induced hyponatraemia so as to promptly take the appropriate measures to face these events.

Our results show that the highest RR of all-grade hyponatraemia was associated with brivanib (RR = 6.5), whilst the highest RRs of high-grade hyponatraemia were reported by brivanib (RR = 5.2), sorafenib (RR = 2.4) and vorinostat (RR = 2.1). Moreover, grouping the selected agents into drug categories even strengthens these data. Indeed, the RR of developing high-grade hyponatraemia with anti-angiogenic agents was 2.69 compared to anti-EGFR TKIs or mAbs.

Recent studies demonstrated the activity of vascular endothelial growth factor (VEGF) in renal sodium metabolism, thus suggesting an activity of anti-VEGF/VEGF receptor agents in the homeostasis of sodium. Gu *et al*. evaluated the correlation between VEGF inhibition and hypertension. They found that rats receiving semaxanib (SU5416), a small-molecule inhibiting VEGF downstream signaling, showed increased mean arterial pressure and natriuresis. They also described a right shift with a slightly higher intercept of the pressure-natriuresis curve in rats with dietary salt-induced hypertension [35]. In addition, Grisk and colleagues reported that early hypertension induced by anti-VEGFR-TKI sunitinib is probably related to the direct action of this agent on the collecting ducts, suggesting a role for VEGFR-TKI in regulating renal sodium reabsorption [36].

Although the exact mechanisms underlying the increased incidence of hyponatraemia in patients exposed to targeted agents is still unclear, these evidences suggest an important role of VEGF/VEGFR pathway in sodium homeostasis. Most of the studies included in this analysis concerned hepatocellular carcinoma. In this condition, risk of developing hyponatremia is increased due also to concomitant liver cirrhosis that stimulates arginine vasopressin (AVP) secretion [37].

This study has several limitations. First of all it is a meta-analysis achieved starting from clinical trials and not from individual patients’ data. This implies also the potential presence of confounding factors that were not considered, for instance patient comorbidities, previous administration of cytotoxic chemotherapy, and simultaneous treatments. In particular, in some of aforementioned trials, target agents were administrated with cisplatin. In this respect, literature data reported evidences about hyponatremia due to cisplatin-based chemotherapy, suggesting two possible mechanisms: stimulation of hypothalamic AVP production and damage of renal tubules with development of salt wasting syndrome [1,38]. Available data are insufficient to exclude a synergic effect between chemotherapy and target agents.

Furthermore, it should be consider that patients eligible for clinical trials mostly show fair organ functions, for this reason the incidence and severity of hyponatraemia may appear underrated in our meta-analysis comparing to clinical practice. Another limitation is the lack of data regarding delays, interruptions and discontinuations because of hyponatraemia, to correlate to our results.

In face of the limitations described, this meta-analysis, for the first time in literature, pointed out a correlation between targeted agents, in particular anti-angiogenetic ones, and hyponatraemia of all- and high-grade in patients with solid tumors. Moreover this study was the first to analyze RR of hyponatraemia in different groups of targeted agents, showing that maximum incidence of hyponatraemia was observed in patients treated with anti-VEGFR agents. Considering the negative prognostic and predictive role of hyponatraemia in cancer patients, a careful and prompt recognition of this event is preferred so as to limit negative consequences on patient outcome and to prevent possible treatment delays or interruptions.

Physicians and patients should be informed of such risks and an appropriate laboratory monitoring should be suggested to early detect hyponatraemia and optimize the management of these agents.

## Conclusion

Hyponatraemia represents a negative prognostic factor for cancer patients. Increasing evidences showed a significant increased risk of developing hyponatraemia in patients treated with biological therapy. Therefore an accurate and frequent monitoring of serum sodium should be evaluated in patients treated with new-targeted agents, in particular with antiangiogenetic drugs, both in clinical practise and in prospective studies, for a rapid diagnosis and correction of this electrolyte disturbance.

## Supporting Information

S1 Fig Funnel graphs for the assessment of potential publication bias among selected studies for all-grade (A) and high-grade (B) hyponatraemia.
(TIF)

S2 Fig PRISMA Checklist. (DOC)
