# Supplementary figures and images for: Risk of Hyponatraemia in Cancer Patients Treated with Targeted Therapies: A Systematic Review and Meta-Analysis of Clinical Trials
Source: PLoS One. 2016 May 11;11(5):e0152079. doi: 10.1371/journal.pone.0152079 (PMC4864354; doi:10.1371/journal.pone.0152079)

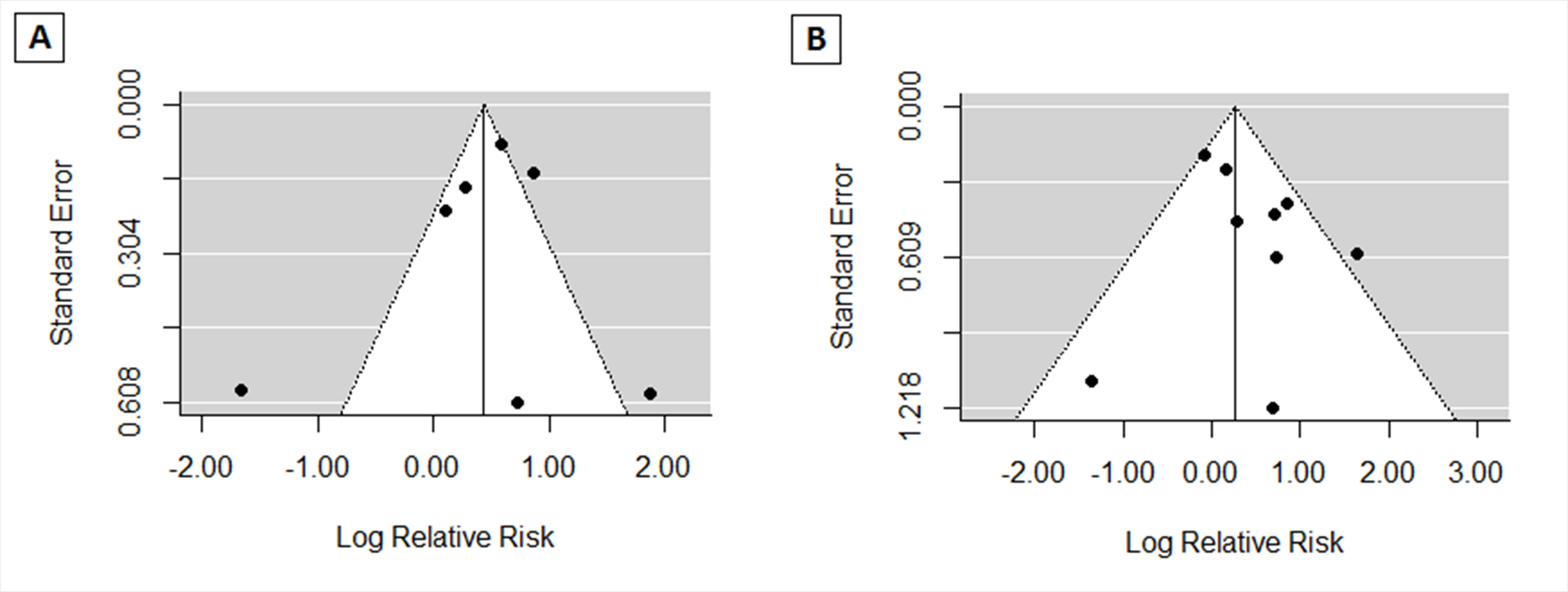

Supplement: S1 Fig — Funnel graphs for the assessment of potential publication bias among selected studies for all-grade (A) and high-grade (B) hyponatraemia. (TIF) [file pone.0152079.s001.tif]
