# Supplementary material for: Risk of Hyponatraemia in Cancer Patients Treated with Targeted Therapies: A Systematic Review and Meta-Analysis of Clinical Trials
Source: PLoS One. 2016 May 11;11(5):e0152079. doi: 10.1371/journal.pone.0152079 (PMC4864354; doi:10.1371/journal.pone.0152079)
Supplement: S2 Fig — (DOC) [file pone.0152079.s002.doc]

| **Section/topic** | **#** | **Checklist item** | **Reported on page #** |
| --- | --- | --- | --- |
| **TITLE** | | |  |
| Title | 1 | **RISK OF HYPONATRAEMIA IN CANCER PATIENTS TREATED WITH TARGETED THERAPIES: A SYSTEMATIC REVIEW AND META-ANALYSIS OF CLINICAL TRIALS.** | 1 |
| **ABSTRACT** | | |  |
| Structured summary | 2 | Background: Hyponatraemia has been reported with targeted therapies in cancer patients. The aim of this study was to perform an up-to-date meta-analysis in order to determine the incidence and relative risk (RR) of hyponatremia in cancer patients treated with these agents.  Materials and Methods: The scientific literature regarding hyponatraemia was extensively reviewed using MEDLINE, PubMed, Embase and Cochrane databases. Eligible studies were selected according to PRISMA statement. Summary incidence, RR, and 95% Confidence Intervals were calculated using random-effects or fixed-effects models based on the heterogeneity of selected studies.  Results: 4803 potentially relevant trials were identified: of them, 13 randomized phase III studies were included in this meta-analysis. 6670 patients treated with 8 targeted agents were included: 2574 patients had hepatocellular carcinoma, whilst 4096 had other malignancies. The highest incidences of all-grade hyponatraemia were observed with the combination of brivanib and cetuximab (63.4) and pazopanib (31.7), while the lowest incidence was reported by afatinib (1.7). The highest incidence of high-grade hyponatraemia was reported by cetuximab (34.8), while the lowest incidences were reported by gefitinib (1.0). Summary RR of developing all-grade and high-grade hyponatraemia with targeted agents was 1.36 and 1.52, respectively. The highest RRs of all-grade and high-grade hyponatraemia were associated with brivanib (6.5 and 5.2, respectively). Grouping by drug category, the RR of high-grade hyponatraemia with angiogenesis inhibitors was 2.69 compared to anti-Epidermal Growth Factor Receptors agents (1.12).  Conclusion: Treatment with biological therapy in cancer patients is associated with a significant increased risk of hyponatraemia, therefore frequent clinical monitoring should be emphasized when managing targeted agents. | 3 |
| **INTRODUCTION** | | |  |
| Rationale | 3 | Targeted therapies interfere with specific molecules involved in cancer cell growth, angiogenesis and survival, in contrast with traditional chemotherapy, drugs that mainly act against all actively dividing cells.  Such a different mechanism of action explains the absence of adverse events traditionally observed with cytotoxic chemotherapy and the occurrence of new drug-related toxicity profiles.  Among serum electrolytes disorders, hyponatraemia is probably the most frequent biochemical alterations potentially related to the use of these new agents. | 4 |
| Objectives | 4 | The objective of the present study was to thoroughly assess incidence and relative risk of hyponatraemia in patients with solid tumors receiving targeted therapies through a revised meta-analysis of clinical trial in literature. | 4 |
| **METHODS** | | |  |
| Protocol and registration | 5 | This systematic review and meta-analysis was achieved adhering to PRISMA guidelines for clinical trial selection in order to assess whether there is a correlation between hyponatraemia and treatment with targeted therapy. | 4 |
| Eligibility criteria | 6 | We evaluated exclusively human studies in English literature that met the requirements listed below: (1) prospective randomized phase III trials enrolling patients affected by solid tumors; (2) patients randomly assigned to treatment arm (targeted agents) or control arm (standard of care, best supportive care or placebo) and (3) provided records regarding treatment-related and non-tumor associated hyponatraemia.  Full articles were obtained, and we checked for additional appropriate references. Where results were reported or updated in two or more publication, we selected the most recent or most thorough.  Studies not specifically addressing the association between treatment with targeted therapy and hyponatraemia were excluded from the analysis. Comparative trials presenting targeted agents in both study arms were not considered, as well as numerous meta-analyses conducted in similar settings. | 5 |
| Information sources | 7 | PubMed from January 1966 – January 31, 2015  EMBASE from 1974 – January 31, 2015  Cochrane from 1967 – January 31, 2015  The principal source of information was derived from published articles. | 4 |
| Search | 8 | Searches were conducted entering combination of the keywords “cancer” or “solid tumor” associated to any of the following words: “abiraterone”, “afatinib”, “aflibercept”, “axitinib”, “bevacizumab”, “brivanib”, “cabozantinib”, “cediranib”, “cetuximab”, “crizotinib”, “dabrafenib”, “dovitinib”, “enzalutamide”, “erlotinib”, “everolimus”, “figitumumab”, “gefitinib”, “icotinib”, “imatinib”, “ipilimumab”, “lapatinib”, “linifanib”, “neratinib”, “nilotinib”, “nivolumab”, “orteronel”, “panitumumab”, “panobinostat”, “pazopanib”, “pembrolizumab”, “pertuzumab”, “ramucirumab”, “regorafenib”, “sorafenib”, “sunitinib”, “T-DM1”, “temsirolimus”, “tivozanib”, “trastuzumab”, “tremelimumab”, “vandetanib”, “vemurafenib”. | 5 |
| Study selection | 9 | We hand-searched bibliographies of retrieved papers for additional references. The selection process of studies is represented in Figure 1. Baseline features of included trials are listed in Table 1. | 7 |
| Data collection process | 10 | Data extraction was conduced from full texts of eligible articles, by two independent evaluators (MS and EN). Data collected comprehended author name list, year of publication, number of enrolled and treated patients, treatment arms characteristic and targeted agent employed, number and grade (all-grade and high grade) of hyponatraemia events reported in each arm.  National Cancer Institute’s Common Terminology Criteria for Adverse Events (CTCAE) version 2 or 3 were used to define adverse events (AEs). Study quality and appropriateness of randomization, double-blinding, and withdrawals was fixed by Jadad scoring system | 5-6 |
| Data items | 11 | The primary endpoint of the present study was to evaluate incidence and relative risk of hyponatraemia in patients with solid tumors receiving targeted therapies. | 4 |
| Risk of bias in individual studies | 12 | Quality of the studies was assessed using the Cochrane criteria. | 9-10 |
| Summary measures | 13 | We considered the following summary measurements: incidence, relative risk (RR), and their corresponding 95% confidence intervals (CIs). Incidence evaluation was performed extracting from the safety section of eligible studies the following data: (1) number of patients receiving targeted therapy and (2) number of hyponatraemia cases. RRs of hyponatraemia was analyzed basing on data extracted from comparative trials in which patients were randomly assigned to receive targeted therapy or controls. | 6 |
| Synthesis of results | 14 | Our results show that the highest RR of all-grade hyponatraemia was associated with brivanib (RR=6.5), whilst the highest RRs of high-grade hyponatraemia were reported by brivanib (RR=5.2), sorafenib (RR=2.4) and vorinostat (RR=2.1). Moreover, grouping the selected agents into drug categories even strengthens these data. Indeed, the RR of developing high-grade hyponatraemia with anti-angiogenic agents was 2.69 compared to anti-EGFR TKIs or mAbs. | 10 |

Page 1 of 2

| **Section/topic** | **#** | **Checklist item** | **Reported on page #** |
| --- | --- | --- | --- |
| Risk of bias across studies | 15 | Publication biases were quantified by Egger and Begg tests for both the incidence and RR. | 9-10 |
| Additional analyses | 16 | Cochran’s Q test was applied to all variables to detect statistical heterogeneity among study outcomes; inconsistency of effects was measured using the I2 index as a parameter of inconsistency across studies attributable to heterogeneity and chance. Homogeneity of variance was violated for p values <0.1. Basing on Cochran’s Q statistical significance it was decided whether to use random effects model (in case of significant Q test) or fixed effects model (in case of not significant Q test). For each variable, model estimate and null hypothesis of overall non-significant difference between study- and control-group were tested. Microsoft Excel 2010 was employed to collect data; data analysis has been carried out with the “MATLAB and Statistics Toolbox Release 2012b”. | 6 |
| **RESULTS** | | |  |
| Study selection | 17 | Four thousand, eight hundreds and three clinical trials studying target treatments employ in neoplastic patients resulted hypothetically significant to our research; of those, 2914 studies did not meet inclusion criteria due to any of the following causes: duplicate, phase I trials, not focused on targeted agents, reviews, observational studies, meta-analyses, case reports, letters or commentaries.  Of the remaining studies, 1658 were non-randomized phase II trials while 218 lacked Drug-related hyponatraemia records in the safety profile. Eventually 13 trials were judged suitable and relevant for our study. | 7 |
| Study characteristics | 18 | Study population accounted for 6670 participants of whom 2574 (39% of the total) were affected by hepatocellular carcinoma (HCC), and 4096 affected by other malignancies (882 patients had gastric cancer, 790 patients had melanoma, 745 had colorectal cancer, 617 had non-small cell lung cancer (NSCLC), 435 had renal cell carcinoma (RCC), 369 had head and neck tumors (HN) and 258 had oesophageal cancer). An Eastern Cooperative Oncology Group performance status (ECOG-PS) not higher than 2 was required for enrolment in all 13 studies along with fair renal and hepatic functions, coagulation and haematological parameters. Baseline features of included trials are listed in Table 1. | 7 |
| Risk of bias within studies | 19 | For a more conservative approach, results of random effect models were presented. | 4-6 |
| Results of individual studies | 20 | RR of all-grade and high-grade hyponatraemia in the overall population and by single study  RR analysis was conducted considering 4 studies for the analyses of all-grade events and 9 for high-grade events. In three studies placebo was administered in the control arm, while patients in the other studies were assigned to active control arms.  In the overall study population, RR of all-grade and high-grade hyponatraemia was 1.36 (95% CI 1.06 to 1.75) for patients receiving targeted treatments compared to 1.52 (95% CI 1.06 to 2.20) in control arms. The RRs of all-grade hyponatraemia across selected trials are reported in Figures 2 and 3.  Eight different agents were available for this analysis. In patients stratified by single studies, the highest RR of all-grade hyponatraemia was associated with brivanib (6.5, 95% CI 2.1 to 21.0). On the other hand, the lowest RR was associated with cetuximab (1.1, 95% CI 0.73 to 1.70).  High-grade hyponatraemia highest RRs occurred with brivanib (5.2; 95% CI 1.6 to 17.0), sorafenib [28] (2.4; 95% CI 1.1 to 5.1) and vorinostat (2.1; 95% CI 0.63 to 0.81), while the lowest RR of high-grade hyponatraemia was observed with cetuximab (0.93; 95% CI 0.63 to 1.42) and gefinitib (0.26; 95% CI 0.03 to 2.33]. The RRs of all-grade hyponatraemia across selected trials are reported in Figures 2 and 3. | 8-9 |
| Synthesis of results | 21 | From patients receiving target therapy, the incidences of all-grade and high-grade hyponatraemia were 25.6% (95% CI 23.8 to 27.4) and 10.0% (95% CI 9.1 to 11.0), respectively.  Maximum incidence of all-grade hyponatraemia was reached with the combination of brivanib and cetuximab (63.4%, 95% CI 58.5 to 68.3) and with pazopanib (31.7%, 95% CI 26.3 to 37.1), the lowest with afatinib (1.7%, 95% CI 0.0 to 3.0). The highest and lowest incidences of high-grade hyponatraemia were observed with cetuximab (44.8%, 95% CI 32.0 to 57.6), and gefitinib (1.0%, 95% CI 0 to 2.3), respectively (table 2).  RR of all-grade and high-grade hyponatraemia was 1.36 (95% CI 1.06 to 1.75) for patients receiving targeted treatments compared to 1.52 (95% CI 1.06 to 2.20) in control arms (figure 2 – 3). In patients stratified by single studies, the highest RR of all-grade hyponatraemia was associated with brivanib (6.5, 95% CI 2.1 to 21.0), the lowest RR with cetuximab (1.1, 95% CI 0.73 to 1.70).  High-grade hyponatraemia highest RRs occurred with brivanib (5.2; 95% CI 1.6 to 17.0), sorafenib (2.4; 95% CI 1.1 to 5.1) and vorinostat (2.1; 95% CI 0.63 to 0.81), the lowest was observed with cetuximab (0.93; 95% CI 0.63 to 1.42) and gefinitib (0.26; 95% CI 0.03 to 2.33].  Grouping target therapy into 2 categories, inhibitors of angiogenesis and anti-Epithelial growth factor receptor angents. The incidence of high-grade hyponatraemia was 7.2 [95% CI 5.5 to 8.8] with the inhibitors of angiogenesis and 8.8 [95% CI 6.9 11.0] with anti-EGFR agents. Moreover, the RR of high-grade hyponatraemia with inhibitors of angiogenesis was 2.69 (95% CI 1.62 to 4.48) compared to anti-EGFR TKIs or mAbs (1.12 95% CI 0.81 to 1.53). | 7-8-9 |
| Risk of bias across studies | 22 | Egger test showed z = 1·62 p = 0·11, while Begg test showed Kendall's tau = 0.1111 p = 0.7614. Funnel plots are showed in Figure S1. | 9-10 |
| Additional analysis | 23 | Occurrences of hyponatraemia (all-greade and high-grade) accounted for a total of 1402 cases; of those 575 all-grade events occurred among the 3036 patients belonging to treatment groups versus 284 among controls. Considering only patients assigned to receive targeted therapy, the incidences of all-grade and high-grade hyponatraemia were 25.6% (95% CI 23.8 to 27.4) and 10.0% (95% CI 9.1 to 11.0), respectively.  All-grade hyponatraemia reached its maximum incidence with the combination of brivanib and cetuximab (63.4%, 95% CI 58.5 to 68.3) and with pazopanib (31.7%, 95% CI 26.3 to 37.1), whereas afatinib (1.7%, 95% CI 0.0 to 3.0) showed the lowest incidence of hyponatraemia. However, several studies did report only high-grade events.  Three hundred and fifty six high-grade hyponatraemia occurrences were reported in patients assigned to treatment arms and 187 in the controls. The highest and lowest incidences of high-grade hyponatraemia were observed with cetuximab (44.8%, 95% CI 32.0 to 57.6), and gefitinib [26] (1.0%, 95% CI 0 to 2.3), respectively. The incidences of all-grade and high-grade hyponatraemia are reported in Table 2.  For an experimental examination, 5 out of the 6 targeted therapies studied in the RR analysis of high-grade hyponatraemia were grouped into 2 categories: (1) inhibitors of angiogenesis (brivanib, pazopanib, sorafenib); (2) anti-Epithelial growth factor receptor tyrosine kinase inhibitors (EGFR-TKIs) or monoclonal antibodies (mAbs) (gefitinib, cetuximab). The same analysis was not performed for all-grade events due to the smaller number of studies available. Afatinib was omitted in reason of an active control arm. Vorinostat was excluded due to the number of patients in this study, which was too small to constitute a single group.  A total of 946 patients received inhibitors of angiogenesis, whereas 442 received anti-EGFR TKIs or mAbs. The incidence of high-grade hyponatraemia was 7.2 [95% CI 5.5 to 8.8] with the inhibitors of angiogenesis and 8.8 [95% CI 6.9 11.0] with anti-EGFR TKIs or mAbs. Moreover, the RR of high-grade hyponatraemia with inhibitors of angiogenesis was 2.69 (95% CI 1.62 to 4.48) compared to anti-EGFR TKIs or mAbs (1.12 95% CI 0.81 to 1.53) (Figure 4). | 8-9 |
| DISCUSSION | | |  |
| Summary of evidence | 24 | Hyponatraemia represents an increasingly important issue in oncology clinical practice since it negatively correlates with performance status and with prognosis of cancer patients. Patients with hyponatraemia have a higher risk of mortality and present a longer time of hospitalization with consequent cost increases. An early detection and a prompt treatment of this disorder could prevent serious neurologic complication and improve overall survival (OS). For this reasons, it is pivotal for both physicians and patients to be aware about the risk of drug-induced hyponatraemia so as to promptly take the appropriate measures to face these events. | 10-11-12 |
| Limitations | 25 | In face of the limitations described, this meta-analysis, for the first time in literature, pointed out a correlation between targeted agents, in particular anti-angiogenetic ones, and hyponatraemia of all- and high-grade in patients with solid tumors. | 11 |
| Conclusions | 26 | Hyponatremia represents a negative prognostic factor for cancer patients. Increasing evidences showed a significant increased risk of developing hyponatraemia in patients treated with biological therapy. | 12 |
| **FUNDING** | | |  |
| Funding | 27 | No separate funding was necessary for the undertaking of this systematic review and meta-analysis.  Rossana Berardi received consulting fee or honoraria from Otsuka, Lilly, Italfarmaco; Stefano Cascinu received consulting fee or honoraria from Lilly, Amgen, Roche and they have received a research funding (paid to their Institution) from BMS for a research project. The other authors declare that they have no competing interests. All authors contributed to the study, read and approved the final manuscript. |  |
